# An open‐label, randomized crossover study to evaluate the acceptability and preference for contraceptive options in female adolescents, 15 to 19 years of age in Cape Town, as a proxy for HIV prevention methods (UChoose)

**DOI:** 10.1002/jia2.25626

**Published:** 2020-10-09

**Authors:** Katherine Gill, Anna‐Ursula Happel, Tanya Pidwell, Andrea Mendelsohn, Menna Duyver, Leigh Johnson, Landon Meyer, Catherine Slack, Ann Strode, Eve Mendel, Lauren Fynn, Melissa Wallace, Hans Spiegel, Heather Jaspan, Jo‐Ann Passmore, Sybil Hosek, Dionne Smit, Alex Rinehart, Linda‐Gail Bekker

**Affiliations:** ^1^ Desmond Tutu HIV Centre University of Cape Town Cape Town South Africa; ^2^ Institute of Infectious Diseases and Molecular Medicine Faculty of Health Sciences University of Cape Town Cape Town South Africa; ^3^ Centre for Infectious Diseases Epidemiology and Research School of Public Health and Family Medicine University of Cape Town Cape Town South Africa; ^4^ Division of Epidemiology and Biostatistics School of Public Health and Family Medicine University of Cape Town Cape Town South Africa; ^5^ HIV AIDS Vaccines Ethics Group University of KwaZulu‐Natal Durban South Africa; ^6^ Cancer Association of South Africa Johannesburg South Africa; ^7^ Department of Health and Human Services Kelly Government Solutions Contractor to National Institute of Allergy and Infectious Diseases National Institutes of Health Rockville MD USA; ^8^ Departments of Pediatrics and Global Health University of Washington Seattle WA USA; ^9^ Centre for Global Infectious Disease Research Seattle Children’s Research Institute Seattle WA USA; ^10^ National Health Laboratory Service (NHLS) Cape Town South Africa; ^11^ Stroger Hospital of Cook County Chicago IL USA; ^12^ MSD (Pty) Ltd Halfway House South Africa; ^13^ ViiV Healthcare Research Triangle Park NC USA

**Keywords:** HIV prevention; youth; South Africa, preference, adolescent girls, adherence

## Abstract

**Introduction:**

Young women in Southern Africa have extremely high HIV incidence rates necessitating the availability of female‐controlled prevention methods. Understanding adolescent preference for seeking contraception would improve our understanding of acceptability, feasibility and adherence to similar modes of delivery for HIV prevention.

**Methods:**

UChoose was an open‐label randomized crossover study over 32 weeks which aimed to evaluate the acceptability and preference for contraceptive options in healthy, HIV‐uninfected, female adolescents aged 15 to 19 years, as a proxy for similar HIV prevention methods. Participants were assigned to a contraceptive method for a period of 16 weeks in the form of a bi‐monthly injectable contraceptive, monthly vaginal Nuvaring^®^ or daily combined oral contraceptive (COC) and then asked to state their preference. At 16 weeks, participants crossed over to another contraceptive method, to ensure that all participants tried the Nuvaring^®^ (least familiar modality) and additionally, either the injection or COC. Primary outcomes were contraceptive acceptability and preference. At the end of the 32 weeks they were also asked to imagine their preference for an HIV prevention modality. Secondary endpoints included changes in sexual behaviour, contraceptive adherence and preference for biomedical and behavioural HIV prevention methods.

**Results:**

Of the 180 participants screened, 130 were enrolled and randomized to the Nuvaring^®^ (n = 45), injection (n = 45) or COC (n = 40). Significantly more Nuvaring^®^ users (24/116; 20.7%) requested to change to another contraceptive option compared to injection (1/73; 1.4% *p* = 0.0002) and COC users (4/49; 8% *p* = 0.074). Of those that remained on the Nuvaring^®^, adherence was significantly higher than to COC (*p* < 0.0001). Significantly more injection users (77/80; 96.3%) thought this delivery mode was convenient to use compared to Nuvaring^®^ (74/89; 83.1%; *p* = 0.0409) or COC (38/50; 76.0%; *p* = 0.0034). Overall, the preferred contraceptive choice was injection, followed by the ring and lastly the pill.

**Conclusions:**

Adherence to daily COC was difficult for adolescents in this cohort and the least favoured potential HIV prevention option. While some preferred vaginal ring use, these data suggest that long‐acting injectables would be the preferred prevention method for adolescent girls and young women. This study highlights the need for additional options for HIV prevention in youth.

## 1 INTRODUCTION

South Africa has the highest burden of HIV in the world with a national prevalence of 18.9% [1, 2, 3]. Adolescent girls and young women account for 29% of all new HIV infections in South Africa [4, 5]. These young women are disproportionally at risk of acquiring HIV because of increased biological susceptibility and gender‐based inequality [6]. Recognizing the importance of adolescent HIV prevention, UNICEF has set a global target of reducing HIV infections in young women by 75% by 2020 [7, 8, 9].

Negotiating safer sex is challenging for young women [10], highlighting the need for female‐controlled HIV prevention methods. Oral PrEP is licensed and becoming available in South Africa [11], whereas the dapivirine vaginal ring [12] and long‐acting injectable cabotegravir [13, 14] are being developed as additional PrEP delivery options. Together, these three HIV prevention methods could potentially provide sexually active women a choice of HIV prevention medications that closely mirror their contraceptive options.

Previous studies, mostly done in adult women have suggested that delivery in a long‐lasting injection would be a good target for drug development [15, 16, 17, 18]. However, given the distinct developmental, physical and social differences between adult women and female adolescents [19], it is essential to explore how the mode of delivery of an HIV prevention option impacts acceptability and use in the adolescent population. Understanding adolescent contraception preferences could assist policymakers in predicting product acceptability and use and will focus development efforts on HIV prevention methods that are most likely to be acceptable to adolescents. Assessing preference based on hypothetical use can be challenging in an adolescent population. We therefore speculated that, if adolescents tested licensed contraception methods that mirrored HIV prevention modalities, it would allow them to explore and predict the acceptability, feasibility and adherence to similar modes of delivery for HIV prevention. This was particularly important in the case of the contraceptive vaginal ring, which although a licensed contraceptive, is not available in the public sector and thus relatively unknown in South Africa.

To help inform PrEP development strategies we designed a crossover study for adolescent girls aged 15‐ 19 that aimed to assess the acceptability, preference and adherence to three contraceptive options – monthly vaginal ring (Nuvaring^®^), bi‐monthly injection and daily combined oral contraceptive pills (COC). These options were selected to emulate the delivery modes of three antiretroviral‐based HIV prevention modalities in use or under development namely oral PrEP, the vaginal ring and long‐acting injectable PrEP.

## 2 METHODS

### 2.1 Study design

The UChoose study was an open‐label, randomized crossover study of healthy, sexually active, HIV‐negative female adolescents aged 15 to 19 years. The study was approved by the Division of AIDS and the University of Cape Town (UCT) Health Science Research Ethics Committee and was conducted in full compliance with South African Good Clinical Practice (SA‐GCP) and ICH‐GCP guidelines and registered in the public registry database of ClinicalTrials.gov (NCT02404038). All participants and their parents/legal guardians (if participant <18 years) provided written informed consent and assent (if participant <18 years) before undergoing any trial‐related procedures.

### 2.2 Setting

The Cape Town based study site is situated within a peri‐urban low‐income community with a high prevalence of HIV. It has a well‐established relationship with community stakeholders, experienced study staff, adolescent friendly sexual and reproductive health services, as well as an active adolescent community advisory group. Recruitment took place through community outreach at the local school, clinic and youth groups.

### 2.3 Study participants

Participants agreed to use a randomly assigned contraceptive method for the duration of the study (32 weeks). Participants were excluded if they were pregnant, living with HIV, or had medical contraindications to study products. Sexually transmitted infections (STIs) diagnosed through testing at screening were treated prior to enrolment. Bacterial vaginosis (BV) was treated syndromically according to the South African treatment guidelines.

### 2.4 Study procedures

**Figure 1:**
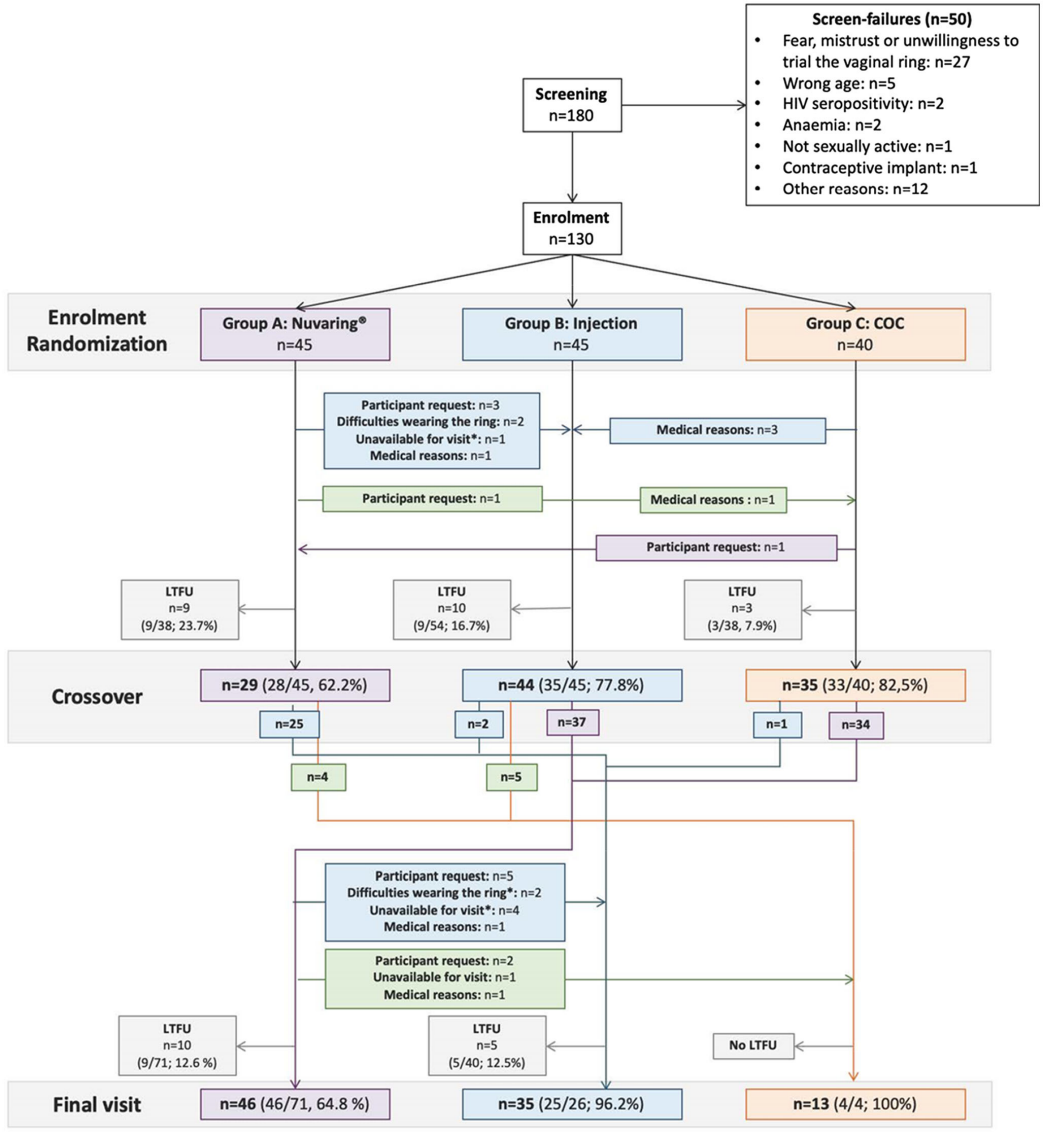
Study overview and randomization. Participants were randomized at enrolment to Nuvaring^®^ (group A), injection (group B) or combined oral contraceptives (COC, group C). The figure indicates how many participants changed to another study arm before the crossover visit, at which all participants from group B and C were switched to Nuvaring, while participants who were previously in group A could choose their subsequent contraceptive method. LTFU, Lost to follow up. N/a, not available for ring change. *Participant was LTFU after study product change.

Eligible participants were randomly assigned in a 1:1:1 ratio to one of three study arms: (A) monthly vaginal Nuvaring^®^ (etonogestrel 11.7 mg/ethinyl estradiol 2.7 mg), (B) bi‐monthly injectable contraceptive (Norethisterone enantate 0.2 g/mL) or (C) daily COC pills (Levonorgestrel 0.15 mg/Ethinyl estradiol; 0.03 mg) for a 16‐week period (Figure 1). After 16 weeks, participants were “crossed over” and those in arms B and C received the Nuvaring^®^ for another 16 weeks, whereas those in arm A were allowed to choose between the injectable and COC. Participants attended follow‐up visits every eight weeks throughout the duration of the study. The study design ensured that all participants would use the less familiar vaginal ring for at least one 16‐week period. Participants received general contraceptive education and counselling and HIV risk reduction counselling at every visit. Participants also received education about ring insertion techniques and were given the option of self‐insertion or having a study clinician place the ring.

### 2.5 Outcomes

The primary outcomes were acceptability and preference of the Nuvaring^®^ versus the injectable and COC. Acceptability was quantified by assessing the rates of premature contraceptive discontinuation and change to another contraceptive method. The reason for changing contraception methods was documented. Contraceptive method acceptability and preference were also measured using the ORTHO birth control satisfaction assessment (BC‐SAT) [20] at crossover and study exit. Participants were required to complete the tool for each method used. The ORTHO BC‐SAT is a standardized tool, developed by Colwell et al, for use among women who utilize hormonal contraceptives to understand which factors contribute to overall contraceptive acceptability and user satisfaction. The ORTHO BC‐SAT consists of eight identified domains: ease of use, compliance, lifestyle impact, symptom/side effect bother, menstrual impact, future fertility concerns, assurance/confidence and overall satisfaction. All multi‐item scales reported acceptable test–retest reliability (0.79 to 0.87). Preference was measured using a detailed interviewer‐assisted questionnaire administered at the final study visit. This questionnaire examined product preference from participant’s personal use of oral tablets, injections and vaginal ring.

Secondary endpoints included contraceptive continuation and adherence, sexual behaviour, safety of the administered study products and preference of biomedical and behavioural HIV prevention methods. Continuation was defined as attending the study visit and receiving the study drug. Adherence was measured by self‐reported use of the study drug. Safety was measured by monitoring laboratory and clinical adverse events using the DAIDS table for grading the severity of adult and paediatric adverse events, version 1.0 December 2004 (clarification August 2009). Sexual behaviour was evaluated by assessing the self‐reported number of sexual partners and condom use, in detailed interviewer‐assisted questionnaires. Preference for biomedical and behavioural HIV prevention methods was measured at study exit using a modified ORTHO‐HP‐Sat questionnaire (modified from the BC‐Sat to assess preference for prevention tools based on their experiences with contraceptive methods).

Laboratory Testing: Pregnancy testing and HIV testing were performed at screening, enrolment and weeks 8, 16, 24 and 32. HIV rapid testing was done in series using a third‐generation Determine^™^ HIV‐1/2 Combo and Uni‐Gold™ Recombigen^®^ HIV‐1/2 for confirmation. STI testing was done at screening, week 16 and the exit visit. HSV‐2 testing (KALON) was performed on blood and a vulvo‐vaginal swab was collected and tested for *C. trachomatis*, *N. gonorrhoea*, *T. vaginalis* and *M. genitalium* using a Multiplex PCR. A vulvo‐vaginal swab was collected for BV testing (Nugent scoring; BV negative (Nugent 0 to 3), intermediate (Nugent 4 to 6) or positive (Nugent 7 to 10)) and Candidiasis screening (*Candida* hyphae and spores). Vaginal pH was measured using colour‐fixed indicator strips (Macherey‐Nagel, Düren, Germany). STI prevalence was measured as the proportion of participants with STIs at the screening, crossover (week 16) and exit (week 32) visits. STI incidence rates were calculated as the number of new infections per person – year. New infections were defined as any infection present that was occurring for the first time or had been treated at a previous study visit. All STIs were treated by study clinicians when results became available.

### 2.6 Data analysis

Data were analysed using Stata version 12.0 (Stata Corporation, College Station, Texas, USA) and RStudio. Graphs were generated using Prism^®^ Version 6 (GraphPad Software, USA). Planned description of continuous variables with means, medians, standard deviations and proportions, as appropriate were calculated. Categorical variables were described as proportions and 95% confidence intervals. All analyses were based on two‐sided statistical tests at alpha = 0.05. Cross‐sectional differences in study population characteristics were tested using Pearson´s Chi‐squared test or Fisher’s exact test (when the expected value was <5). Unpaired Student’s t‐test was used to test differences in means and unpaired Mann–Whitney U test was applied for differences in medians. McNemar’s test was used for paired longitudinal nominal data.

## 3 RESULTS

### 3.1 Participants and baseline characteristics

Between September 2015 and July 2017, 180 participants screened and 130 of these enrolled (Figure 1). The most common reason for ineligibility was unwillingness to be randomized and to use the vaginal ring (27/50, 54.0%), incorrect age (5/50, 10.0%), HIV sero‐positivity (2/50, 4.0%), anemia (2/50, 4.0%), not being sexually active (1/50, 2.0%) and having the contraceptive implant (1/50, 2.0%). Twelve participants (12/180, 6.7%) did not enrol for social reasons, including relocation and family or peer pressure not to join. Table 1 summarizes cohort characteristics at enrolment. Three quarters (75%) of participants were living with one or both parents. The median age was 17 years (IQR 16 to 18) and the majority (112/129; 86.8%) were attending school.

**Table 1:** Demographics, reported sexual behaviour, pregnancy history at baseline

		Overall (n = 130)	Arm A Nuvaring^®^ 45/130; 34.6%		Arm B Injection 45/130; 34.6%	Arm C COC 40/130; 30.8%
Age [years, median (IQR)]		17 (16 to 18)	17 (16 to 18)		17 (16 to 18)	17 (16 to 18)
Living with parents [n/N (%)]		98/130 (75%)	33/45 (73%)		34/45 (76%)	31/40 (78%)
Use of alcohol in preceding 12 months [n/N (%)]		80/130 (61.5%)	26/45 (57.8%)		34/45 (75.6%)	20/40 (50.0%)
Age at menarche [years, median (IQR)]		13 (12 to 14)	13 (12 to 14)		13 (12 to 14)	13 (12 to 14)
Education [n/N (%)]						
School attendance		112/129 (87%)	38/45 (84%)		39/45 (87%)	36/40 (90%)
Highes grade completed		10 (8 to 11)	9 (8 to 10)		10 (9 to 11)	10 (8 to 11)
Tertiary attendance		4/120 (3%)	2/44 (5%)		0/45 (0.0%)	2/40 (5.0%)
Sexual behaviour						
Age of sexual debut [median (IQR)]		15 (14 to 16)	15 (14 to 16)		15 (14 to 16)	15 (14 to 16)
Number Sexual partners past year [median (IQR)]		1 (1 to 1)	1 (1 to 1)		1 (1 to 2)	1 (1 to 1)
Multiple sexual partners past year [n/N (%)]		12/130 (9%)	4/45 (9%)		5/45 (11%)	3/40 (8%)
Partner had multiple sexual partners past year [n/N (%)]		27/130 (21%)	9/45 (20%)		10/45 (22%)	8/40 (20%)
New sexual partners past year [n/N (%)]		36/130 (28%)	12/45 (27%)		15/45 (33%)	9/40 (23%)
Number sex acts/week [median (IQR)]		1 (1 to 2)	1 (1 to 2)		1 (1 to 2)	1 (1 to 2)
Condom use at last sexual act [n/N (%)]		66/130 (51%)	24/45 (53%)		19/45 (42%)	23/40 (58%)
Intergenerational (≥5 years age difference) sex [n/N (%)]		26/130 (20%)	8/45 (18%)		10/45 (22%)	8/40 (20%)
Transactional sex [n/N (%)]		1/130 (1%)	0/45 (0%)		1/43 (2%)	0/40 (0%)
Anal sex [n/N (%)]		4/130 (3%)	4/45 (9%)		0/43 (0.0%)	0/40 (0%)
Felt she was at high risk (≥ 70%) of acquiring HIV [n/N (%)]		7/130 (5%)	2/45 (4%)		4/45 (9%)	1/40 (3%)
Felt she had high level of protection (≥ 70%) against acquiring HIV [n/N (%)]		93/130 (72%)	30/45 (67%)		33/45 (73%)	30/40 (75%)
Previously pregnant [n/N (%)]		17/130 (13%)	6/45 (13%)		7/45 (16%)	4/40 (10%)
Prior contraceptive use [n (%)]						
Never	5 (3.8%)		2 (4.4%)	2 (4.4%)		1 (2.5%)
Not currently	26 (20.0%)		6 (13.3%)	10 (22.2%)		10 (25.0%)
Injection (NET‐EN or DMPA)	88 (67.7%)		33 (73.3%)	28 (62.2%)		27 (67.0%)
COC	6 (4.6%)		3 (6.7%)	2 (4.5%)		1 (2.5%)
Implant	3 (2.3%)		1 (2.2%)	2 (4.5%)		0 (0%)

### 3.2 Study overview

Of the enrolled participants (n = 130), 45 were randomized to arm A (Nuvaring^®^), 45 to arm B (injection) and 40 to arm C (COC). At cross‐over, 71 were then switched to the Nuvaring^®^, and of those previously using the ring, 28/45 chose injection and 9/45 chose to change to the COC. Thus, at study completion, 116 participants had used the Nuvaring^®^, 73 the injection and 48 COC. One hundred and eight participants (83.1%) participants attended the crossover visit, and 94/130 (74.6%) participants completed all follow‐up visits (Figure 1). Three participants were prematurely discontinued from the study, two participants no longer wanted contraception and one became pregnant (Nuvaring^®^ user). The most common reason for lost‐to‐follow‐up (LTFU) was missing more than three consecutive study visits (n = 19), choosing not to participate anymore (n = 7), relocating (n = 5) or declining to use the Nuvaring^®^ after randomization (n = 2).

Injectables were the most popular method of contraception prior to enrolment with 88/130 (67.7%) using either Depo Provera or Nuristerate, whereas only 5.6% (6/130) had used COC and none had previously used vaginal rings. Almost a fifth of the participants (17/130) had been pregnant at least once prior to enrolment. Only 3.8% (5/130) of participants were hormonal contraception‐naïve at enrolment (but 20% (26/130) of the cohort had not used contraception within the last three months prior to trial commencement.

The median age at sexual debut was 15 years (IQR 14 to 16). About one‐fifth (21%; 27/130) reported age‐disparate relationships (>5 years difference) and half of the participants reported that they had not used a condom at their last sex act (49%; 64/130). Ten percent (12/130) of participants reported multiple partners. Two‐thirds (61.6%; 80/130) reported a history of alcohol use.

### 3.3 Sexually transmitted infections

Almost half (42.0%; 55/130) of the participants tested positive for at least one curable STI at screening, with the most common STI being *C. trachomatis* (43/130; 33%) (Table 2).

**Table 2:** Prevalence of STIs, BV and candida according to study visit

	Baseline	Crossover	Exit
	n = 130	n = 107	n = 92
Any curable STI	55/130 (42%)	30/107 (28%)	26/92 (28%)
*C. trachomatis*	43/130 (33%)	16/107 (15%)	17/92 (19%)
*N. gonorrhoea*	13/130 (10%)	10/107 (9%)	6/92 (7%)
*T. vaginalis*	12/130 (9%)	3/107 (3%)	3/92 (3%)
*M. genitalium*	3/130 (2%)	5/107 (5%)	2/92 (2%)
HSV‐2 serology	39/130 (30%)	37/107 (35%)	34/92 (37%)
BV (Nugent 7 to 10)	57/130 (44%)	43/107 (40%)	37/92 (40%)
Yeast	20/130 (15%)	17/107 (16%)	21/92 (23%)
Any condition	105/130 (81%)	76/107 (71%)	70/92 (76%)

Bacterial vaginosis (BV) was also frequently observed (44.0%). There was one incident HIV infection in a participant, randomized to receive the injection, giving an HIV incidence of 1.57 per 100 person‐years (95% CI: 0.0779 to 7.68). The HSV‐2 incidence was 26 per 100 person‐years (95% CI: 19 to 38) over the course of the study (Table 3).

**Table 3:** STI incidence rate (confidence interval)

STI	Incidence rate: infections/100 person years
*Chlamydia trachomatis*	84/100 person years (95% CI: 64 to 110)
*Neisseria gonorrhoea*	50/100 person years (95% CI: 34 to 70)
*Herpes Simplex Virus‐2*	26/100 person years (95% CI: 19 to 38)
*Mycoplasma genitalium*	11/100 person years (95% CI: 4 to 22)
*Trichomonas vaginalis*	9/100 person years (95% CI: 3 to 20)
HIV	1.57/100 person years (95% CI 0.8 to 7.68)

At study exit, 37% of the participants tested positive for HSV‐2. Despite self‐reported condom use remaining consistent with on average 50% of participants reporting condom use at last sex act at all study visits, the prevalence and incidence of bacterial STIs remained high throughout the study with 23% of participants treated for a curable STI at crossover and 26% treated at study exit. Similarly, the prevalence of BV remained high throughout the trial. Importantly, the prevalence of BV (Nugent score 7 to 10), candida and STIs were similar between all arms at all visits suggesting that the randomly assigned contraceptive arm did not appear to impact BV or STI risk. (Table 2).

### 3.4 Primary outcome – contraceptive acceptability

Of the 116 participants who were randomized to the Nuvaring^®^ over the course of the study, 24 (20.7%) chose to change to another contraceptive, with the most common reasons being participant request (n = 11), being unavailable for a study visit and unwilling to change the ring by themselves (n = 6) and difficulties in wearing the ring (n = 7) (Figure 1). By contrast, only one participant randomized to the injection (1/73; 1.4%; *p* = 0.0002) changed contraceptive method early and this was due to injection refusal. Four (4/49, 8.1%; *p* = 0.0748) participants randomized to COC changed to another arm, due to medical reasons (headache, nausea and breastfeeding) (Figure 1). In the first 16‐week period, individuals were randomly assigned to a contraceptive whilst some element of choice was allowed in the second period. When reviewing data (not shown) for each period separately the same patterns of switch by method were seen in both periods.

While participants reported that they found all methods equally convenient, a significantly higher number of COC users expressed dissatisfaction because they struggled to remember to use the daily pill (35/50, 70%) compared to injection (16/80; 20.0%; *p* = 0.0004) and Nuvaring^®^ users (15/89; 17%; *p* < 0.0001) users. Similar proportions of adolescents in all three arms were worried about pregnancy, whereas significantly more injection users (37/80; 46.3%) were concerned that the injection would cause future fertility issues, than Nuvaring^®^ (24/89; 27.0%: *p* < 0001) and COC (11/50; 22.0%; *p* < 0.002) users. However, significantly more injection users would recommend the birth control method to a friend (72/80; 90.0%) in comparison to COC users (27/50; 54.0%; *p* < 0.0001) or Nuvaring^®^ users (61/89, 68.5%; *p* = 0.0052). Injection users were also more willing to continue using injectables over other methods (injection [47/80, 58.8%] vs. Nuvaring^®^ [31/89, 34.8%] *p* = 0.0056; injection vs. COC [21/50, 42.0%] *p* = 0.1876) and were extremely satisfied overall (injection [44/80, 55.0%] vs. COC [14/50, 28.0%] *p* = 0.0073; injection vs. Nuvaring^®^ [33/89, 37.1%]. *p* = 0.0556). Overall, Nuvaring^®^ was the second most acceptable method and COC the least popular. Irrespective of the preferred method, adolescents rated ease of use, protection from pregnancy, absence of side effects, being familiar with the method and ease of remembering as the most important factors to be considered, whereas enjoyable sex and a longer dosing regimen did not appear as important.

### 3.5 Primary outcome: contraceptive preference

**Figure 2:**
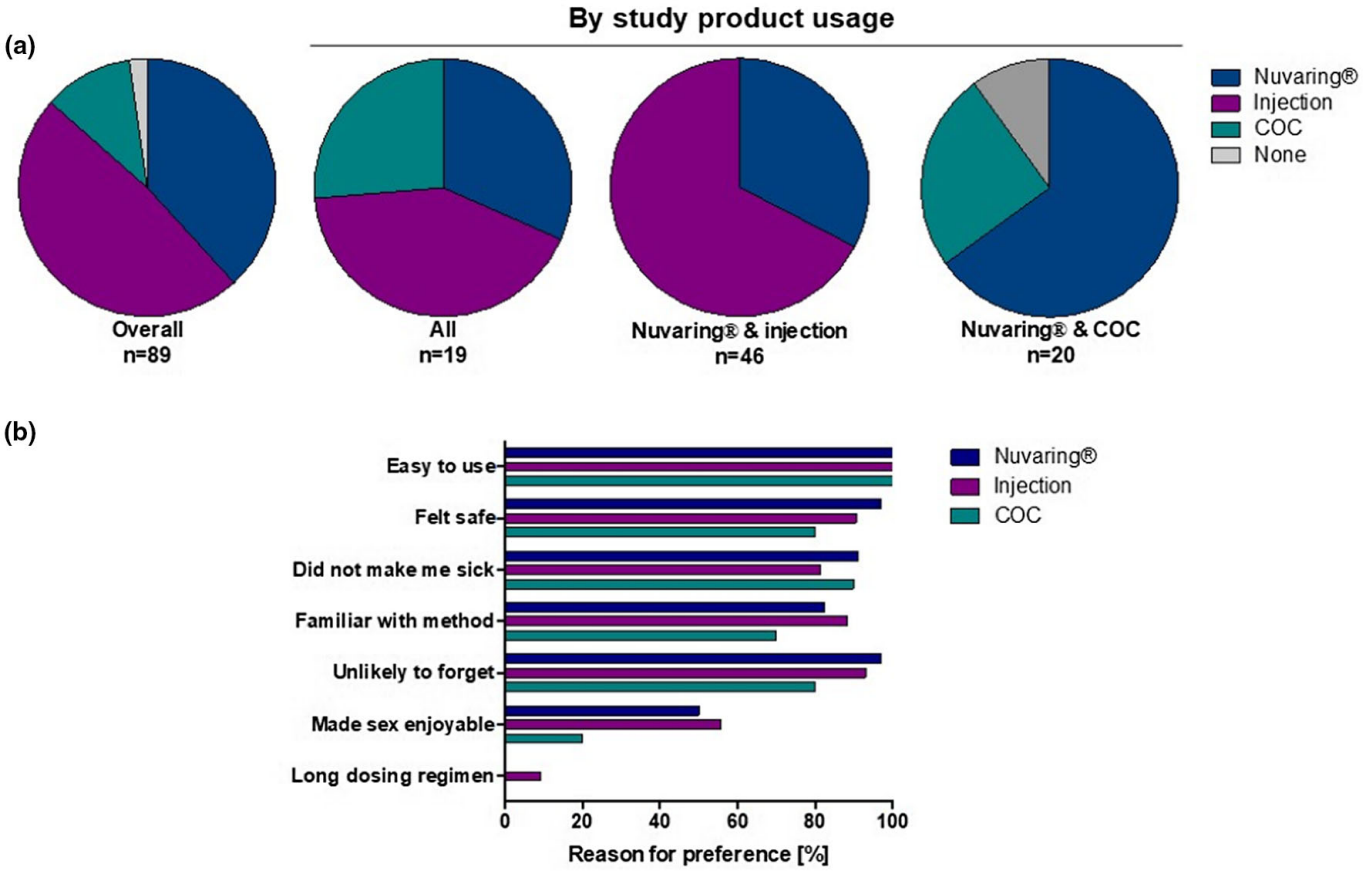
Contraceptive product preference. **(A)** Overall reported preference (n = 89) and reported preference by participants who used all study products (n = 19), Nuvaring^®^ and injection (n = 46), or Nuvaring^®^ and COC (n = 20). **(B)** Percentage of participants within one preference group (Nuvaring ^®^ n = 34, injection n = 43 or COC n = 10) reporting reasons why they preferred this specific product are displayed in the graph.

At the final study visit, 89 participants completed a questionnaire assessing contraceptive preference (Figure 2).

Overall, participants preferred the injection (n = 43, 48%) to Nuvaring^®^ (n = 34, 38%) and COC (n = 10, 11%)) as a contraceptive method, whereas two participants preferred none of the methods (Figure 2). Of the 46 participants who used the Nuvaring^®^ and the injection during the study, the majority preferred the injection (31/46; 67.4%), whereas the majority of participants who used the Nuvaring^®^ and COC preferred the Nuvaring^®^ (13/20, 65.0%).

### 3.6 Secondary outcomes – safety, adherence and HIV prevention product preference

#### 3.6.1 Safety

There were no serious adverse events (AEs) noted on this trial and no AEs were more severe than grade 2. Only 15% of reported AEs were related to study product use and included already recognized side effects of hormonal contraceptives, such as abnormal uterine bleeding, headaches, mastalgia and weight gain.

#### 3.6.2 Adherence

**Figure 3:**
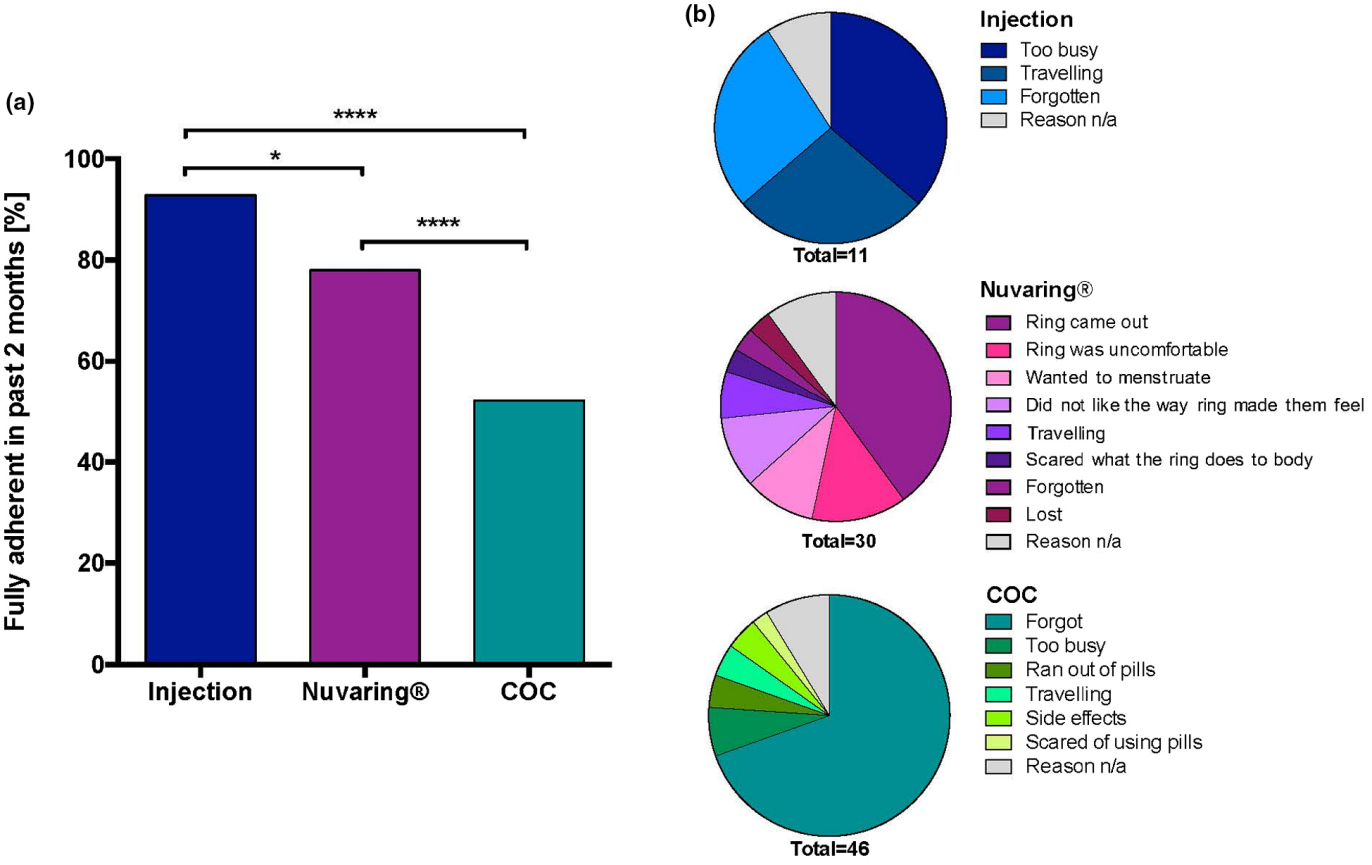
Contraceptive adherence. **(A)** Self‐reported full adherence in the previous two months by injection (n = 54), Nuvaring (n = 173) and COC (n = 96) users. **(B)** Self‐reported reasons for non‐adherence by injection users (n = 11) in the last 2 months and Nuvaring (n = 30) and COC (n = 46) in the previous week.

At each follow‐up visit, participants completed a self‐reported questionnaire to assess adherence to the contraceptive they had used in the previous eight weeks (Figure 3).

As expected, injection users (n = 154 questionnaires completed) reported complete adherence in the majority of the cases (143/154; 92.8%), whereas 78% of Nuvaring^®^ users (n = 173 questionnaires completed) reported to have used the Nuvaring^®^ exactly as instructed in the past two months. In contrast, significantly fewer COC users (n = 96 questionnaires completed) reported full adherence in the previous eight weeks. (50/96; 52.1%; *p* < 0.0001). The most common self‐reported reasons for non‐adherence among Nuvaring^®^ users were that the “ring came out” (12/30; 40.0%) or discomfort (4/30; 13.3%). In contrast the main reason among COC users for non‐ adherence was forgetting to take the pill (32/46; 69.6%). Self‐reported reasons for better adherence were protection from pregnancy, study participation and support by peers and family, regardless of contraceptive method used.

### 3.7 HIV prevention product preference

When asked about preference for potential HIV prevention methods, the majority of participants chose an injectable option (41/89; 46.1%), followed by a vaginal ring (33/89; 37.1%), then a pill (9/89; 10.1%, *p* < 0.0001 vs. injection & vaginal ring), condoms (4/89; 4.5%; *p* < 0.0001 vs. injection & vaginal ring), no penetrative sex (1/89; 1.1%; *p* < 0.0001 vs. injection & vaginal ring) and monogamy (1/89; 1.1%;. *p* < 0.0001 vs. injection & vaginal ring). It was noteworthy that none of the participants chose regular HIV testing or a topical vaginal gel as their preferred method. Overall an injectable option or a vaginal ring were the preferred HIV prevention methods.

### 3.8 DISCUSSION

The UChoose study is the first randomized controlled crossover trial in adolescent South African women evaluating adolescent preference for contraceptive options as a proxy for HIV prevention methods.

Extrapolating from their contraceptive experience South African adolescents preferred an injectable biomedical HIV prevention method, due to its ease of administration and long‐lasting effects, followed by a vaginal ring and lastly oral PrEP. More than two‐thirds of the cohort had used oral or injectable contraception prior to the study, whereas no participants had used the contraceptive ring. Reticence towards using the Nuvaring^®^ may be attributed to a lack of familiarity with vaginal products or a lack of belief that a ring could prevent pregnancy or HIV. Non‐PrEP related forms of HIV prevention (condoms, abstinence, HIV testing) were less popular than PrEP options in this young sexually active cohort.

There was high inter‐individual variability amongst preferences, underscoring the need for a menu of HIV prevention options, including oral PrEP, vaginal rings, male and female condoms, as well as regular testing and injectable PrEP when available. These results are consistent with the findings of other studies of product preference conducted in young African women [21, 22, 23]. It is also likely that these needs and preferences might change over time as adolescents’ transition to young adulthood. Changing preferences may be related to many factors including relationship stability, medical history ability to negotiate condoms with different partners and perception of risk. Easy transitions from one option to another would be ideal. It is possible that the efficacy of a product in the prevention of HIV may also affect decision making when these products become available as other studies have reported that highly effective products are more important than other attributes [21, 22, 23].

HIV incidence was relatively low in this young cohort over a short follow‐up period. All available risk reduction interventions were offered at every visit including STI treatment and regular HIV testing and regular risk‐reduction counselling. Although oral PrEP was not included because it was not yet available as standard of care for HIV prevention in South Africa during this study [24], all other available risk reduction interventions were offered at every visit including regular HIV testing and risk‐reduction counselling. STI prevalence at baseline was high and STI incidence remained high throughout the study, despite treatment and referral for partner treatment. This along with self‐reported high‐risk sexual behaviour, emphasize the need to prevent both STIs and pregnancy adolescent females through integrated adolescent friendly STI and HIV prevention, sexual health and contraceptive services.

It was noteworthy that the greatest motivator for contraceptive adherence was participants’ desire to protect themselves from pregnancy. It is concerning that despite their high‐risk sexual activity and South Africa’s high HIV burden, adolescents perceived themselves to be at low risk for acquiring HIV. This echoes findings from other studies conducted in Africa that have shown similar low‐risk perception [25, 26, 27]. Further work is needed to explore the persistent discordance between adolescent perceived risk and their actual risk. Given that preventing pregnancy is already a highly motivating adherence factor for adolescent females, the development of novel multipurpose technologies that simultaneously prevent HIV and pregnancy might ultimately be the best way to meet the sexual and reproductive health needs of this population. Based on contraception preference, we argue that an injectable HIV prevention drug would likely be the most popular method of PrEP if all three options, oral, injectable, and the ring, were available for use.

We acknowledge that this study had several limitations. The proposed sample size (n = 150) was not reached due to difficulties in participant recruitment. The reasons for this included the need for parental proxy consent for study enrolment since the study involved minors and participants’ unwillingness or reluctance to use the unfamiliar Nuvaring^®^. As reported here, when ring familiarity was attained, the ring desirability increased. All sexual risk behaviour and adherence data were self‐reported and thus subject to bias. Adherence to assigned contraceptive method was supported by study staff including bringing participants back for ring changes and placement checks between visits. Low rates of reported barrier contraceptive use meant that participants who were thought to be at risk of pregnancy due to non‐adherence to their randomized method were changed by study staff early in the study to another contraceptive method, resulting in lower exposure to the Nuvaring^®^. The relatively high lost to follow‐up rate may have introduced bias into the study results.

Our experience here, and that of the recently completed dapivirine ring trials, suggests that women and particularly young women may need time to familiarize themselves with new products [28]. Belief in a novel intervention is complex and influenced by multiple factors including community perceptions, peer, parent and partner support. Hormonal side effects may have also confounded participants’ choice of method and the equivalent side effects would not be present with HIV prevention interventions. Making placebo/non‐active variations of products to be tried by the end user would be an alternative way to give end user’s experience in order to inform their choices. The TRIO study conducted in South Africa using placebo injections, rings and pills also found that injections were the most liked and best‐used products in young women [29].

Given that the study involved an adolescent population and unintended pregnancy would be considered an adverse outcome, it was felt that enabling contraceptive adherence as much as possible was an important ethical consideration. Individuals were initially randomized to a contraceptive option of oral, injectable or ring but in order to enable better adherence to any contraceptive, a low threshold to allow switch was employed. We required every participant to experience the vaginal ring option since this was the least familiar method and therefore more likely to be theoretical for individuals if not experienced. Participants were supported to adhere to their assigned method but if they were unable to they were changed by the study clinician. At least half of the participants were not using condoms consistently and were at high risk for unwanted pregnancy.

Allowing participants to choose between the injectable and COC after the cross over visit may have introduced bias towards the more preferred method when compared to the Nuvaring and may have resulted in the Nuvaring appearing less acceptable than it was.

## 4 CONCLUSIONS

In conclusion, adherence to daily pills proved difficult for adolescents in this study. While some preferred vaginal ring use, most preferred a long‐acting injectable method for pregnancy and HIV prevention. This choice seems to have been largely motivated by the fear of an inability to consistently adhere to daily regimens. It also suggests that familiarity to a method is an important consideration. This study highlights the importance of allowing young women to have method choice as well as information and support as they navigate their sexual and reproductive health options.

## COMPETING INTERESTS

AR is an employee of Viiv Healthcare, DS is an employee of MSD South Africa. MSD sponsored the Nuvarings. Viiv funded the study.

## AUTHORS’ CONTRIBUTIONS

KG contributed to the design of the study. KG also carried out acquisition, analysis and interpretation of data. She participated in drafting the manuscript and revising it. AH participated in data analysis and interpretation. AH critically revised the manuscript. TP, AM, MD, LF, EM all participated in the acquisition and interpretation of data and critically reviewing the manuscript. LJ and LM contributed to the conception and design of the study and analysis and interpretation of data. LJ and LM critically revised the manuscript. CS and AS contributed to the conception and design of the study and analysis and interpretation of data. CS and AS critically revised the manuscript. MW, SH, HJ and JAP contributed to the conception and design of the study. MW, SH, HJ and JAP critically revised the manuscript. HS contributed to the analysis and interpretation of data and critically revised the manuscript. DS and AR critically revised the manuscript. LGB contributed to the conception and design of the study. LGB also carried out the interpretation of the data and drafting and revising the manuscript. All authors read and approved the final version of the manuscript.
